# Proximal and Distal Parts of Sweetpotato Adventitious Roots Display Differences in Root Architecture, Lignin, and Starch Metabolism and Their Developmental Fates

**DOI:** 10.3389/fpls.2020.609923

**Published:** 2021-01-21

**Authors:** Vikram Singh, Hanita Zemach, Sara Shabtai, Roni Aloni, Jun Yang, Peng Zhang, Lidiya Sergeeva, Wilco Ligterink, Nurit Firon

**Affiliations:** ^1^Department of Vegetable and Field Crops, Institute of Plant Sciences, Agricultural Research Organization, The Volcani Center, Rishon Le-Zion, Israel; ^2^Department of Fruit Tree Sciences, Institute of Plant Sciences, Agricultural Research Organization, The Volcani Center, Rishon Le-Zion, Israel; ^3^School of Plant Sciences and Food Security, Tel Aviv University, Tel Aviv, Israel; ^4^Shanghai Chenshan Plant Science Research Center, Chinese Academy of Sciences, Shanghai Chenshan Botanical Garden, Shanghai, China; ^5^CAS Center for Excellence in Molecular Plant Sciences, Chinese Academy of Sciences, Shanghai, China; ^6^Laboratory of Plant Physiology, Department of Plant Sciences, Wageningen University & Research, Wageningen, Netherlands

**Keywords:** adventitious root development, gene expression, lignin, root anatomy, starch, storage-root, sweetpotato

## Abstract

Sweetpotato is an important food crop globally, serving as a rich source of carbohydrates, vitamins, fiber, and micronutrients. Sweetpotato yield depends on the modification of adventitious roots into storage roots. The underlying mechanism of this developmental switch is not fully understood. Interestingly, storage-root formation is manifested by formation of starch-accumulating parenchyma cells and bulking of the distal part of the root, while the proximal part does not show bulking. This system, where two parts of the same adventitious root display different developmental fates, was used by us in order to better characterize the anatomical, physiological, and molecular mechanisms involved in sweetpotato storage-root formation. We show that, as early as 1 and 2 weeks after planting, the proximal part of the root exhibited enhanced xylem development together with increased/massive lignin deposition, while, at the same time, the distal root part exhibited significantly elevated starch accumulation. In accordance with these developmental differences, the proximal root part exhibited up-regulated transcript levels of sweetpotato orthologs of *Arabidopsis* vascular-development regulators and key genes of lignin biosynthesis, while the distal part showed up-regulation of genes encoding enzymes of starch biosynthesis. All these recorded differences between proximal and distal root parts were further enhanced at 5 weeks after planting, when storage roots were formed at the distal part. Our results point to down-regulation of fiber formation and lignification, together with up-regulation of starch biosynthesis, as the main events underlying storage-root formation, marking/highlighting several genes as potential regulators, providing a valuable database of genes for further research.

## Highlights

– Sweetpotato storage-root formation at the distal part of the adventitious root is associated with down-regulation of fiber formation and lignification, together with higher starch accumulation.

## Introduction

Sweetpotato [*Ipomoea batatas* (L.) Lam., family Convolvulaceae] yield depends on a change in the developmental fate of adventitious roots (ARs) into storage roots (SRs). ARs originated from sweetpotato stem cuttings act as the propagation material (Ma et al., 2015). These ARs form white lignified roots unless primary and secondary cambial cells are formed around AR xylem elements, marking the developmental transition of ARs into SRs (Villordon et al., 2009). During SR formation, the number of such vascular cambial cells increases and they develop into starch-accumulating parenchyma cells (Firon et al., 2013; Singh et al., 2019). However, those ARs that do not become SRs exhibit high stele lignification (Villordon et al., 2009). This issue of SR formation is of high economical and agricultural importance, since sweetpotato is an important food crop globally (CIP, 2017), being a rich source of carbohydrates, vitamins, dietary fiber, and micronutrients, with 112.8 million tons produced during 2017 (FAOSTAT, 2017). The mechanism(s) underlying the developmental switch of ARs, enabling development of either lignified roots or SRs, are still unclear (Singh et al., 2019).

As summarized recently by Singh et al. (2019), initiation of SR formation involves up-regulated expression of genes previously suggested, in numerous systems, to regulate cambial cells, as well as genes involved in starch biosynthesis (Schrader et al., 2004; Scofield and Murray, 2006; Tanaka et al., 2008; Firon et al., 2013). Such genes include *class I knotted 1-like* (*KNOX*) genes, on one hand, as well as *sucrose synthase* (*SuSy*), *phosphoglucomutase* (*PGM*), *ADP glucose pyrophosphorylase* (*AGPase*), *granule-bound starch synthase* (*GBSS*), and *starch phosphorylase* (*SP*), on the other hand, respectively.

In parallel to starch accumulation, SR formation/initiation was found to involve down-regulation of lignin-biosynthetic genes and reduced lignin accumulation (Firon et al., 2013). Moreover, under conditions that inhibit SR formation, e.g., application of the plant hormone gibberellin, significant up-regulation of major lignin-biosynthetic genes and of potential upstream-regulators, together with increased lignin levels, was demonstrated (Singh et al., 2019).

Singh et al. (2019) identified sweetpotato orthologs of various *Arabidopsis* lignin-biosynthesis genes, as well as positive and negative regulators of the lignin-biosynthesis pathway. Lignin-biosynthesis genes include *phenylalanine ammonia-lyase* (*PAL*), encoding the enzyme responsible for deamination of phenylalanine (Boerjan et al., 2003), a reaction followed by a series of reactions involving the following genes/enzymes: *cinnamic acid 4-hydroxylase (C4H)*; *4-coumarate:CoA ligase (4CL)*, *hydroxycinnamoyl-coenzyme A shikimate:quinate hydroxycinnamoyl-transferase (HCT)*, *p-coumaroyl shikimate 3’-hydroxylase (C3’H)*, *caffeoyl CoA 3-O-methyltransferase (CCoAOMT)*, *cinnamoyl-CoA reductase (CCR)*, *ferulate 5-hydroxylase (F5H)*, *caffeic acid/5-hydroxyferulic acid O-methyltransferase (COMT)*, *cinnamyl alcohol dehydrogenase (CAD)*, and *hydroxycinnamaldehyde dehydrogenase (HCALDH)* (Raes et al., 2003). Master regulators of vascular development are upstream regulators of secondary cell wall deposition (lignin, cellulose, and hemicellulose), consisting vascular-related NAC domain (VND) (Ohashi-Ito et al., 2010) and secondary-wall-associated NAC domain protein/NAC secondary-wall thickening promoting factor (SND/NST) (Zhong et al., 2006, 2007). Positive regulators VND5, 6, and 7 allow xylem differentiation into protoxylem and metaxylem, and secondary-wall biosynthesis (Kubo et al., 2005; Yamaguchi, 2008; Zhou et al., 2014), while SND1/NST1 and SND2 were shown to regulate secondary cell wall formation in xylem vessels and xylem fiber differentiation (Zhong et al., 2006; Mitsuda et al., 2007; Hussey et al., 2011). VND-INTERACTING 2 (VNI2) and XYLEM NAC DOMAIN 1 (XND1) negatively regulate xylem formation and differentiation, repress VND7-induced expression of vessel-specific genes, and affect/reduce lignin accumulation (Zhao et al., 2008; Yamaguchi et al., 2010). Sweetpotato orthologs of all these regulatory genes, together with members of the abovementioned *KNOX* gene family, were recently suggested as candidates for regulating the developmental switch of the sweetpotato AR into a SR (Singh et al., 2019).

In the current study, we made use of the interesting observation that SR formation is manifested by development of starch-accumulating parenchyma cells and bulking of the distal part of the AR, while the proximal part (first 3 cm that is close to the stem) does not show bulking. This system, where two parts of the same AR display different developmental fates, is a good platform/experimental setup to be used for studying the mechanisms involved in sweetpotato SR formation. Therefore, we compared the anatomical, physiological, and molecular changes that take place during development of the proximal and distal parts of sweetpotato ARs during 1, 2, and 5 weeks (1W, 2W, and 5W, respectively) after planting.

The results show that, as early as 1W and 2W after planting, there are significant differences in the number of xylem vessels and area occupied by xylem fibers, between the two root parts, with the proximal part exhibiting enhanced xylem development together with increased/massive lignin deposition. At the same time, the distal root part exhibited significantly elevated starch accumulation as compared to the proximal part. In accordance with these developmental differences, the proximal root part exhibited up-regulation of sweetpotato orthologs of *Arabidopsis* vascular-development regulators and of key genes of lignin biosynthesis, while the distal part showed up-regulation of genes encoding enzymes of starch biosynthesis. In addition, up-regulated expression levels of an ortholog of the *Arabidopsis BREVIPEDICELLUS* transcription factor (*IbKN2*; a *KNOX* gene), regulator of meristem maintenance, was observed in the distal, as compared to the proximal, root part, as early as 1W after planting. All these recorded differences between proximal and distal root parts were further enhanced at 5W after planting, when SRs were formed at the distal part. Taken together, the results highlight down-regulation of fiber formation and lignification, together with elevated starch accumulation as the main events underlying SR development and point to potential regulators of this developmental switch.

## Materials and Methods

### Plant Material and Growth Conditions

**FIGURE 1:**
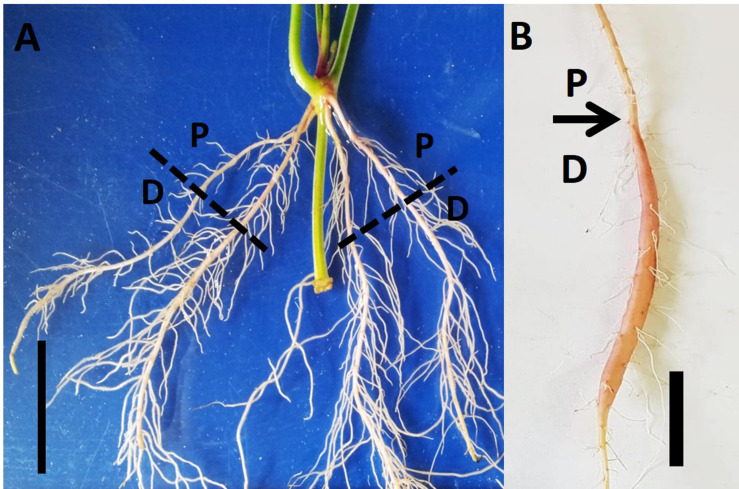
Proximal and distal parts of the Sweetpotato “*Georgia Jet*” adventitious root during development. Root system architecture is presented as recorded at an early phase of root development (2 weeks after planting; **A**) and at 5 weeks after planting when development of a storage root is obvious **(B)**. The proximal (P) and distal (D) root parts represent 0–3 cm and 3–10 cm, respectively, measured from the stem. Scale bar = 3 cm.

Virus-tested stem cuttings of sweetpotato “*Georgia Jet*” cultivar containing three nodes (node numbers 7–9 from shoot apex; Ma et al., 2015) were obtained from the Hasharon region, Israel. Cuttings were planted into PVC pots (dimensions: diameter, 10 cm; length, 30 cm), pre-filled with washed sand. Leaves from node 9 were removed carefully and then node 9 was immersed inside the sand. Plants were allowed to grow in a greenhouse at the Volcani Center, Rishon LeZion, Israel, during May 2017 under temperature conditions of 25/20°C ± 3 day/night temperatures. During the whole experiment, plants were grown under natural conditions without requiring supplemental light. Plants were moistened to half field capacity with water (100 ml) on every third day, until 2W after planting. Thereafter, low N fertilizer solution (100 mg L^–1^ of 20:20:20 N:P:K) was given two times a week, until the end of the experiment. Sampling was done at three time points: 1W, 2W, and 5W after planting using a minimum of 18 plants at each time point. These time points were considered according to the root developmental behavior (i) 2W is the time when roots develop either to lignified roots or SRs (Villordon et al., 2009), while (ii) at 5W SR formation has appeared. At each time point, the whole root system, originating from node 9, was collected from each of the 18 sampled plants. Following harvesting, roots were categorized into two parts: (i) proximal (P; 0–3 cm close to the stem) and (ii) distal (D; 3–10 cm from the stem; Figure 1). The sampled roots, of both categories (P and D), were used for analyzing root system architecture (RSA) parameters, root anatomy, starch levels, and gene expression as detailed below, enabling comparisons between P and D parts, at physiological and molecular levels. The experimental setup is described in Supplementary Figure 1.

### Root System Analysis

Digital imaging was done for the whole root system sampled at 2W and 5W after planting as indicated above, using six plants per sampling time (Supplementary Figure 1). Images were examined by using ImageJ software (ImageJ 1.51a, NIH, United States; Schneider et al., 2012), examining P and D parts of the root separately. The RSA parameters measured included the following: lateral root (LR) number and LR length per plant, and LR density per AR (LR number divided by the respective AR length; 3 and 7 cm, for the P and D parts, respectively).

### Histochemical Analysis and Autofluorescence Imaging

Root samples were taken from six to eight plants at 1W, 2W, and 5W after planting, where 5W samples include both SRs and lignified non-SRs. At each time point (1W, 2W, and 5W) and root type (SRs and lignified non-SRs) the P and D root parts were collected separately (Supplementary Figure 1). All root samples were stored in FAA solution until analysis. The composition of 1 L of FAA solution was 35% formaldehyde (100 ml), glacial acetic acid (50 ml), 96% ethanol (520 ml), and dH_2_O (330 ml).

Histochemical analysis and autofluorescence imaging were done as described by Singh et al. (2019). Samples were first dehydrated using ethanol dilution series, then embedded in paraffin wax for their sectioning using a microtome (Ruzin, 1999). Root sections having a thickness of 15 μm were prepared by using microtome (Leica RM2245, Leica Biosystems, Nussloch, Germany). The root sections were deparafinized using a histoclear solution and then rehydrated with ethanol dilution series. These processed root sections were used for histochemical staining and autofluorescence imaging. The histochemical staining was executed by using safranin-fast green or Phloroglucinol-HCl (Ph-HCl or Weisner) stain to observe the root vascular system and lignin deposition as detailed by Singh et al. (2019). Phloroglucinol-HCl staining was performed according to Mitra and Loque (2014). Microscopic examination of sections was performed by using a light microscope (Leica, Germany) and images were taken by a Nikon DS-Fi1 digital camera. Autofluorescence imaging was performed by using confocal microscopy for unstained and deparaffinized sections (Donaldson and Knox, 2012). All microscopic observations and image acquisitions were performed by a Leica SP8 laser scanning microscope (Leica, Wetzlar, Germany), consisting a solid state laser with 405 nm light, using the Leica Application Suite X software (LASX, Leica, Wetzlar, Germany).

Images captured after histochemical analysis were used for analyzing root vascular parameters. The total xylem vessels were determined by counting the number of protoxylem, metaxylem and secondary xylem. The ImageJ software (ImageJ 1.51a, NIH, United States; Schneider et al., 2012) was used to analyze/calculate the following parameters: total root area, area occupied by xylem elements (vessels and fibers) and percent root area covered by xylem vessels consisting, protoxylem, metaxylem, and secondary xylem.

### Starch Analysis

Roots were sampled from 18 plants at each time point, 1W, 2W, and 5W after planting. The P and D root parts for these samples were collected separately (Supplementary Figure 1). Total 250 mg root sample was grounded into fine power and starch content was determined by the established protocol (Singh et al., 2019). Glucose (0.04%) was used as standard.

### RNA Extraction and Gene Expression Analysis

Roots were sampled from 18 plants at each time point, 1W, 2W, and 5W after planting, collecting the P and D parts separately (Supplementary Figure 1). The root samples were stored immediately at −80°C until analysis. Total RNA extraction and cDNA preparation were done by using RNeasy Plant Mini Kit (Qiagen, Germany) and Verso cDNA Synthesis Kit (Thermo Fisher Scientific, Lithuania), respectively. Quantitative reverse transcriptase-PCR (qRT-PCR) analysis for examining gene expression was performed in a reaction mixture (10 μl) containing cDNA, forward and reverse primers, and ABsolute Blue qPCR SYBR Green ROX Mix (Thermo Fisher Scientific, Lithuania), using a Rotor Gene 6000 Real-Time PCR System (Corbett Life Science, Australia). Reaction conditions were set at 95°C for 10 s, 60°C for 15 s, and 72°C for 20 s, with 40 cycles. The results were analyzed by Rotor gene software, and relative expression of genes was calculated by either 2^–ΔCt^ or 2^–ΔΔCt^ method using Phospholipase D1a (PLD) as a reference gene. Primers were designed using Primer3Plus^1^ (Supplementary Table 1). Heat map was prepared by MultiExperiment Viewer, MeV v4.9 software^2^, using the expression profiles obtained by the 2^–ΔΔCt^ method. The hierarchical clustering of genes was based on the Pearson correlation, which allows genes clustering according to their expression pattern and levels.

### Statistical Analysis

Statistical analysis of data was performed by Student’s *t*-test at *P* ≤ 0.05, using JMP 5.0.1a statistical software (SAS Institute Inc., NC, United States).

## Results

### Proximal and Distal Parts of the Sweetpotato AR Differ With Respect to Root System Architecture Parameters

**FIGURE 2:**
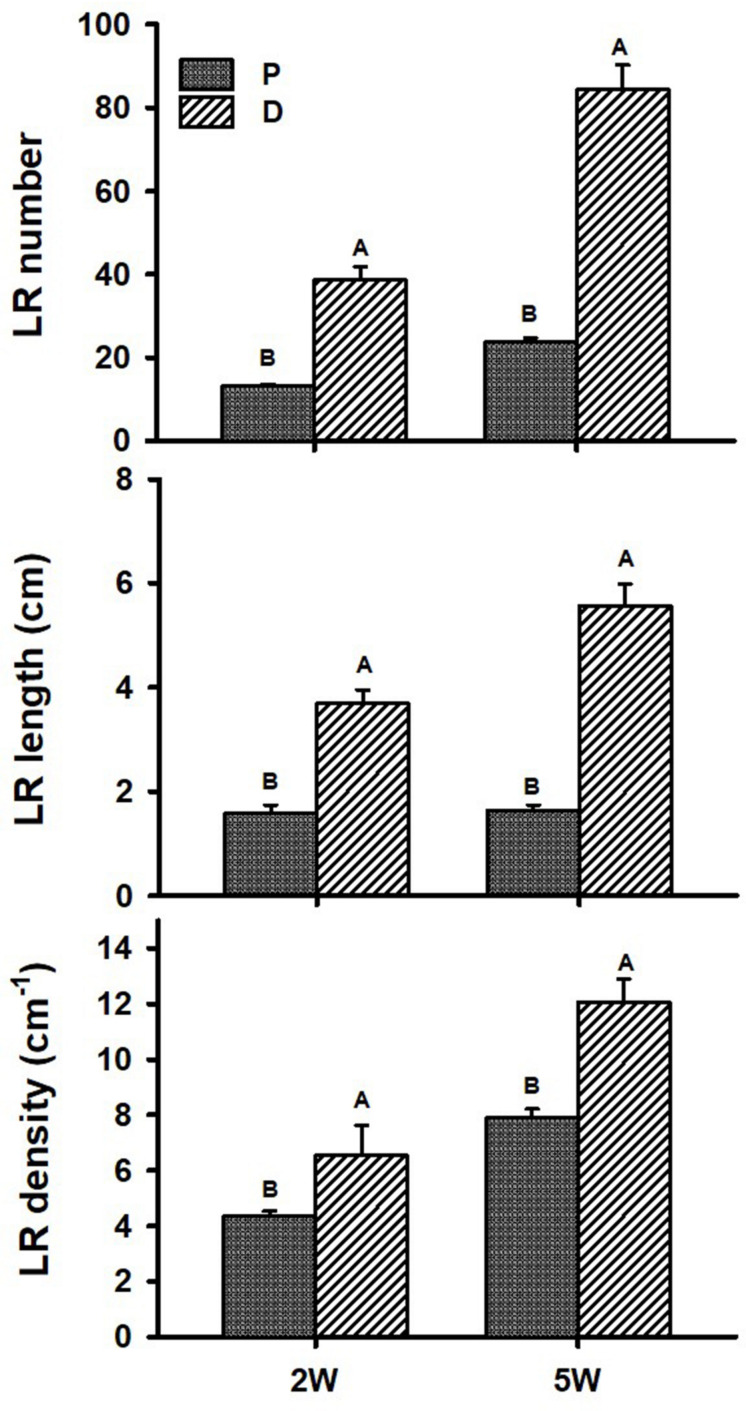
Root system architecture parameters of proximal (P) and distal (D) parts of the Sweetpotato “*Georgia Jet*” adventitious root during development. Proximal and distal root parts were sampled at 0–3 cm and 3–10 cm from the stem, respectively. The parameters included lateral root (LR) number per plant, LR cumulative length per plant, and LR density per adventitious root, recorded at 2 weeks (2W) and 5 weeks (5W) after planting. At 5W, the distal part of the root represents a storage root. Bars represent mean of six independent replicates (plants) ± SE. Significance analysis was performed using Student’s *t*-test (*P* ≤ 0.05), where unlike letters represent significant differences between the two root parts within a sampling group.

Figure 1 illustrates our experimental system, comparing AR characteristics between the proximal (P; first 3 cm close to the stem) and distal (D; 3 to 10 cm from the stem) parts of the root during plant and root development. AR characteristics were studied during the first 2W after planting (the phase in root development during which a developmental decision will be made toward becoming either a SR or a lignified non-SR; Villordon et al., 2009), as well as at 5W after planting (when SR formation is observed/obvious). Differences in RSA parameters, between P and D root parts, were followed and results are presented in Figure 2. The results show a significantly higher number of LRs, higher LR length, as well as higher LR density, recorded in the D as compared to the P part of the root at 2W after planting. Similar results were obtained at 5W after planting, with LR number, length, and density exhibiting more than twofold higher values in the D compared to the P part of the root (Figure 2).

### Sweetpotato Root Xylem Development and Lignification Differ Between the Proximal and Distal Parts

**FIGURE 3:**
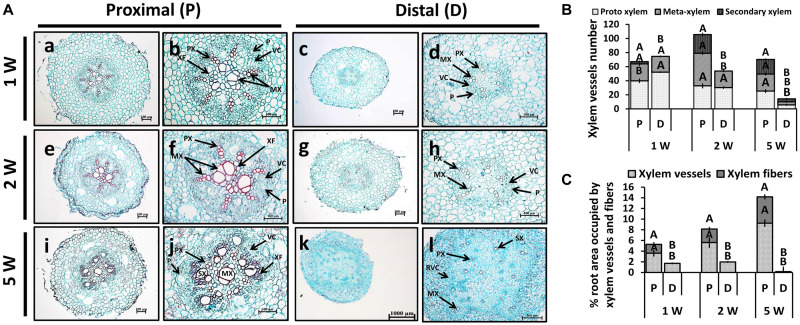
Anatomy of the proximal (P) and distal (D) parts of the Sweetpotato “*Georgia Jet*” adventitious root (AR) during development. Proximal and distal root parts were sampled at 0–3 cm and 3–10 cm from the stem, respectively. Sampling was done at 1, 2, and 5 weeks after planting (1W, 2W, and 5W, respectively). At 5W, the D part of the root represents a storage root. **(A)** Sections were stained with safranin and fast green (showing lignified vessels stained red), and represent six to eight roots sampled from individual plants. PX, protoxylem; MX, metaxylem; SX, secondary xylem; XF, xylem fibers; VC, vascular cambium; RVC, regular vascular cambium; P, phloem. Scale bar = 100 μm (a–j), 1000 μm (k), and 500 μm (l). **(B)** Xylem vessel number in P and D parts of Sweetpotato ARs. Xylem vessel number was evaluated as per AR and included protoxylem, metaxylem, and secondary xylem. **(C)** Percent (%) root area covered by xylem vessels (protoxylem, metaxylem, and secondary xylem) and xylem fibers is shown. The differentiation between primary and secondary xylem is based on the following observation: The primary xylem is built of protoxylem vessels that form a pattern of a star. In the middle of the star pattern, a wide metaxylem vessel or sometimes two metaxylem vessels differentiate in the root’s center. All the fibers are produced by the cambium that differentiate between the primary phloem and the primary xylem. Therefore, the fibers are determined secondary fibers. The secondary xylem consists of the secondary fibers and secondary vessels, both produced by the cambium. Bars in panels **(B,C)** represent mean of six to eight independent replicates (plants) ± SE. Significance analysis was performed by using Student’s *t*-test (*P* ≤ 0.05), where unlike letters represent significant differences between the two root parts within a sampling group.

Adventitious root anatomy and lignin deposition were analyzed using root cross-sections derived from sweetpotato plants at 1W, 2W, and 5W after planting, comparing between P and D parts of the root. The 5W samples included both ARs that developed into SRs and those that did not. The results, presented in Figure 3, show development of significantly higher number of xylem vessels in the P compared to D part of the root, evident at 2W and 5W after planting. At an early phase of root development, 1W and 2W after planting, most vessels were primary xylem (protoxylem and metaxylem; Figures 3A,B). In the P part of the root, the development of xylem fibers was already obvious at 1W and 2W after planting, as evident by the area occupied by fibers (1.7 and 2.6%, respectively), increasing at 5W to 4.9%, while no fibers were detected in the D part of the root (Figure 3C). As detailed in the legend to Figure 3, primary xylem is built of protoxylem vessels that form a pattern of a star. In the middle of the star pattern, a wide metaxylem vessel or sometimes two metaxylem vessels differentiate in the root’s center. All the fibers are produced by the cambium that differentiate between the primary phloem and the primary xylem.

Interestingly, at 5W after planting, there were significant differences in xylem development between the P and D parts of the root, even in those roots that did not develop SRs (Supplementary Figure 2). Namely, twofold higher number of secondary xylem vessels was observed in the P compared to the D root part, and a significantly higher root area was found to be occupied by fibers (Supplementary Figure 2).

**FIGURE 4:**
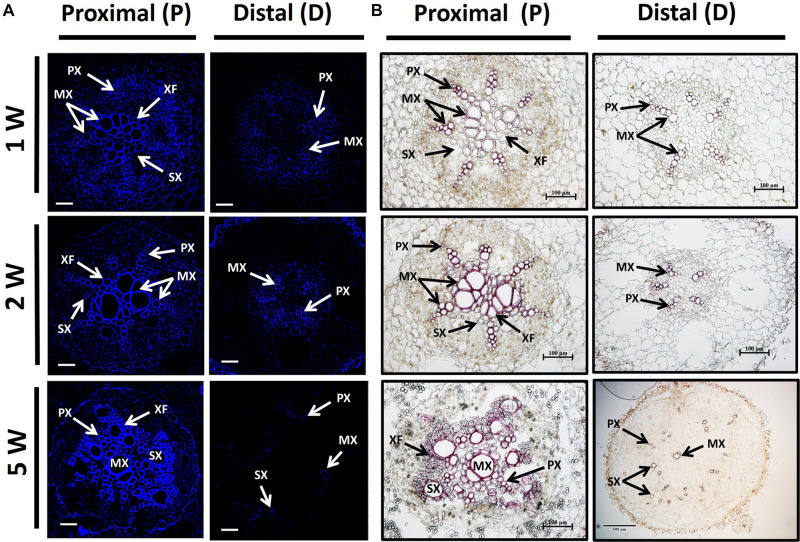
Lignin accumulation in sections of proximal (P) and distal (D) parts of the Sweetpotato “*Georgia Jet*” adventitious root (AR). Proximal and distal root parts were sampled at 0–3 cm and 3–10 cm from the stem, respectively. Representative cross-sections of ARs, sampled at 1, 2, and 5 weeks (1W, 2W, and 5W, respectively) after planting, are presented as viewed by autofluorescence imaging **(A)** and phloroglucinol-HCl staining **(B)**. At 5W, the D root part represents sampled storage roots. Autofluorescence imaging (a non-specific tool) showing secondary-wall material, lignin, and various phenolic structures, as blue fluorescence emission following UV excitation at 365 nm was used to follow lignin accumulation by xylem vessels ( proto-, meta-, and secondary xylem) and xylem fibers **(A)**. Phloroglucinol-HCl staining (being more specific and indicative of 4-O-linked hydroxycinnamyl aldehyde structures in lignins) stains lignified cells wall, where lignin deposition appears as a pink-red color in proto-, meta-, and secondary xylem, and xylem fibers **(B)**. Presented sections represent six to eight roots sampled from individual plants (six to eight replicates). PX, protoxylem; MX, metaxylem; SX, secondary xylem; XF, xylem fibers. Scale bar = 50 μm **(A)**, 100 μm **(B)**.

In order to follow lignin deposition/accumulation in the P and D parts of the root during development, we have used autofluorescence imaging (being a non-specific tool indicative of lignin and different phenolic molecules/structures; Donaldson and Knox, 2012) as well as phloroglucinol-HCl staining (which interacts with the 4-O-linked hydroxycinnamyl aldehyde structures in lignins to develop pink color) (Pomar et al., 2002). The results, presented in Figure 4, indicate higher lignification in root cross-sections of the P part compared to the D part, as visualized by both autofluorescence and phloroglucinol staining (Figures 4A,B, respectively). Highest differences in lignification/lignified area were evident in 5W samples comparing between the P part and the SRs developed in the D part (Figure 4).

### Starch Accumulation in Proximal and Distal Parts of the Sweetpotato AR During Development

**FIGURE 5:**
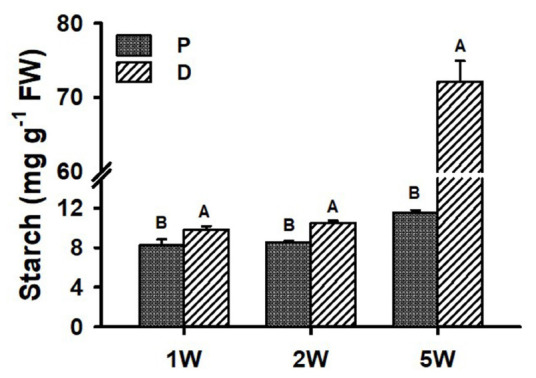
Starch levels in Sweetpotato “*Georgia Jet*” adventitious root proximal (P) and distal (D) parts during development. Roots were sampled at 1, 2, and 5 weeks (1W, 2W, and 5W, respectively) after planting. At 5W, D root part represents sampled storage roots. Bars show mean of three independent biological replicates, where each biological replicate represent roots collected from six independent plants) ± SE. Significance analysis was performed by using Student’s *t*-test (*P* ≤ 0.05), where unlike letters represent significant differences between the two root parts within a sampling group. Storage roots were sampled from the middle part of the storage organ.

Starch accumulation (indicator of SR initiation) was analyzed during ARs development (1W, 2W, and 5W) in both P and D root parts. Interestingly, as early as 1W and 2W after planting, when there were no visible signs yet of SR development/bulking, the D part of the root exhibited significantly higher starch content as compared to the P part of the root (10.5 mg g^–1^ FW compared to 8.5 mg g^–1^ FW at 2W after planting; Figure 5). At 5W after planting, differences between the two parts of the root increased, with the D, SR-forming part, exhibiting 6.2-fold higher starch accumulation.

### Proximal and Distal Parts of the Sweetpotato AR Display/Exhibit Differences in Expression Pattern of Key Genes Involved in Regulation of Vascular Development, Lignin Biosynthesis, and Carbohydrate Metabolism

We have used the following sweetpotato orthologs of *Arabidopsis* genes, involved in regulation of vascular development and identified by us recently (Singh et al., 2019), to follow their expression during root development: *IbNAC075*, *IbVND7*, *IbSND2*, *IbXND1*, *IbVNI2*, and *IbVNI2-like*. These genes/orthologs were identified using the *Georgia Jet* root transcriptome database (Firon et al., 2013) and homology searches that were done using Blast2GO and using the non-redundant NR and the plant TFDB databases^3^. A scheme of the regulatory network of secondary development is presented in Supplementary Figure 3, marking the identified sweetpotato genes. None of the second-level orthologs (*MYB46/83*) or downstream transcription factors (such as *MYB58/63*, for example) were detected, except five MYB4-like orthologs that exhibited very low expression and did not give reproducible results. The list of all sweetpotato contigs with their respective *Arabidopsis* genes is presented in Table 1.

**TABLE 1:** Sweetpotato (*Ipomoea batata)* “*Georgia Jet*” orthologs/genes investigated in our study.

*Ipomoea batata* contigs	*Ipomoea batata* gene	Source/NCBI accessions	*Arabidopsis* homolog	*Arabidopsis* gene name	References	Gene function	Gene Family
**Regulators of vascular development**							
S_PBL_c36855	VASCULAR RELATED NAC-DOMAIN PROTEIN 075 (*IbNAC075*)	Firon et al., 2013	AT4G29230	NAC DOMAIN CONTAINING PROTEIN 75, (*NAC075*)	Endo et al., 2015	Secondary wall biosynthesis in xylem vessels	**NAC DOMAIN PROTEIN**
S_PBL_c32341	VASCULAR RELATED NAC-DOMAIN PROTEIN 7 (*IbVND7*)	Firon et al., 2013	AT1G71930	VASCULAR RELATED NAC-DOMAIN PROTEIN 7 (*VND7*)	Yamaguchi et al., 2008	Xylem vessel differentiation	
S_PBL_c24252	SECONDARY WALL-ASSOCIATED NAC DOMAIN 2 (*IbSND2*)	Firon et al., 2013	AT4G28500	SECONDARY WALL-ASSOCIATED NAC DOMAIN PROTEIN 2, (*SND2*)	Hussey et al., 2011	Secondary wall biosynthesis in xylem fibers	
S_PBL_c504	XYLEM NAC DOMAIN1 (*IbXND1*)	Firon et al., 2013	AT5G64530	XYLEM NAC DOMAIN 1 (*XND1*)	Zhao et al., 2008	Negative regulator of xylem vessel differentiation and secondary wall thickening	
S_PBL_c4628	VND-INTERACTING2 (*IbVNI2*)	Firon et al., 2013	AT5G13180	NAC DOMAIN CONTAINING PROTEIN 83 (*NAC083*), VND-INTERACTING 2 (*VNI2*)	Yamaguchi et al., 2010	Negative regulator of xylem vessel differentiation and secondary wall thickening	
S_PBL_c17476	VND-INTERACTING2 like (*IbVNI2-like*)	Firon et al., 2013	AT5G13180	NAC DOMAIN CONTAINING PROTEIN 83-like (*NAC083*-like), VND-INTERACTING 2 (*VNI2*-like)	Yamaguchi et al., 2010	Negative regulator of xylem vessel differentiation and secondary wall thickening	
**Lignin biosynthesis**							
S_PBL_c2312	Phenylalanine ammonia lyase (*IbPAL*)	Firon et al., 2013	AT2G37040	Phenylalanine ammonia lyase 1 (*PAL1*)	Huang et al., 2010	Lignin biosynthesis	**Various**
S_PBL_c7605	Cinnamate 4-hydroxylase (*IbC4H*)	Firon et al., 2013; GQ373157	AT2G30490	Cinnamate-4-hydroxylase (*C4H*)	Schilmiller et al., 2009	Lignin biosynthesis	
S_PBL_c18044	4-Coumarate-CoA ligase (*Ib4CL*)	Firon et al., 2013	AT1G51680	4-Coumarate:Coa ligase 1 (*4CL*)	Lee et al., 1997	Lignin biosynthesis	
S_PBL_c17752	Hydroxycinnamoyl transferase (*IbHCT*)	Firon et al., 2013; AB576768	AT5G48930	Hydroxycinnamoyl transferase (*HCT*)	Hoffmann et al., 2004	Lignin biosynthesis	
S_PBL_c2944	Caffeoyl-CoA-O-methyltransferase (*IbCCoAOMT*)	Firon et al., 2013; EU250002	AT4G34050	Caffeoyl coenzyme A o-methyltransferase 1 (*CCoAOMT*)	Do et al., 2007	Lignin biosynthesis	
S_PBL_lrc53688	Cinnamyl alcohol dehydrogenase (*IbCAD*)	Firon et al., 2013; GU380306	AT4G39330	Cinnamyl alcohol dehydrogenase (*CAD*)	Sibout et al., 2005	Lignin biosynthesis	
**Class I knotted 1-like**							
S_PBL_c8137	Class I knotted1-like homeobox (KNOX1) (*IbKN2*)	Firon et al., 2013; AB283028	AT4G08150	BREVIPEDICELLUS (*KNAT1*)	Mele et al., 2003	Meristematic maintenance	**KNOX1**
S_PBL_c31412	Class I knotted1-like homeobox (KNOX1) (*IbKN3*)	Firon et al., 2013; AB283029	AT4G08150	BREVIPEDICELLUS (*KNAT1*)	Mele et al., 2003	Meristematic maintenance	
**Carbohydrate metabolism and starch biosynthesis**							
S_PBL_c543	Sucrose synthase (*IbSuSy*)	Firon et al., 2013; EU908020	AT3G43190	Sucrose synthase 4 (*SUS4*)	Angeles-Nunez and Tiessen, 2010	Sucrose metabolism	**Various**
S_PBL_c20112	Phosphoglucomutase (*IbPGM*)	Firon et al., 2013	AT1G70730	Phosphoglucomutase 2 (*PGM2*)	Kofler et al., 2000	Interconversion of glucose 1-phosphate and glucose 6-phosphate	
S_PBL_c18129	ADP-glucose pyrophosphorylase alpha subunit (*IbAGPa1*)	Firon et al., 2013; KJ365312, JQ797696, Z79635, X83498, Z46756, AY544766	AT5G48300	ADP-glucose pyrophosphorylase small subunit	Ventriglia et al., 2008	Starch biosynthesis	
S_PBL_c54187	ADP-glucose pyrophosphorylase beta subunit (*IbAGPb1A*)	Firon et al., 2013; JQ797698, JQ797692, AB271013, AF068260, AJ249257, AJ249256, AJ252316, AJ245392, AB071976	AT1G27680	ADP-glucose pyrophosphorylase large subunit	Ventriglia et al., 2008	Starch biosynthesis	
S_PBL_c3042	Granule-bound starch synthase (*IbGBSS*)	Firon et al., 2013; AB524722/23/24/25/26/27/28, AB071604	AT1G32900	Granule-bound starch synthase 1 (*GBSS1*)	Zeeman et al., 2002	Starch biosynthesis	
S_PBL_c1370	Starch phosphorylase (*IbSP*)	Firon et al., 2013; L25626, M64362	AT3G29320	ALPHA-glucan phosphorylase 1 (*PHS1*)	Malinova and Fettke, 2017	Phosphorolytic degradation of starch	

**FIGURE 6:**
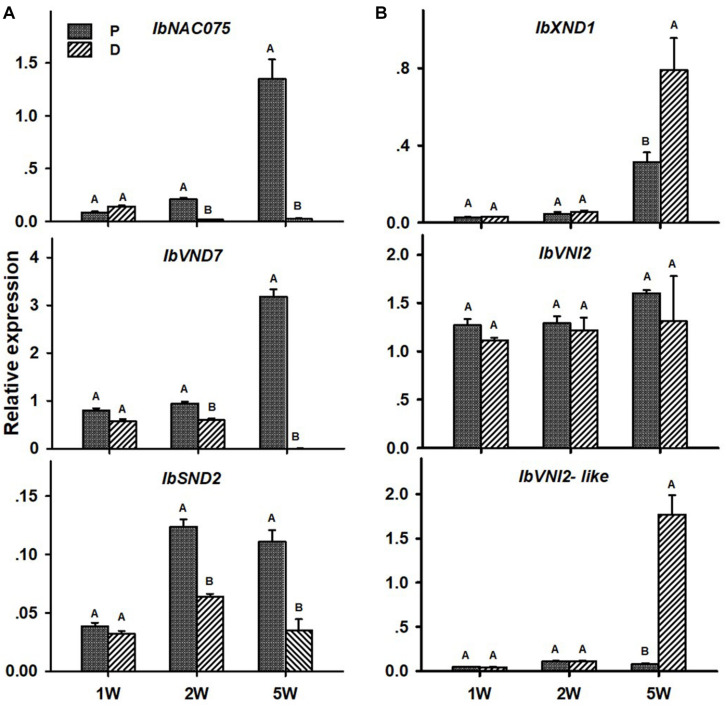
Expression profiles of Sweetpotato “*Georgia Jet*” potential positive **(A)** and negative **(B)** regulators of vascular development in proximal (P) and distal (D) parts of the adventitious root during development. Transcript levels were measured in roots collected at 1, 2, and 5 weeks after planting (1W, 2W, and 5W, respectively). At 5W, roots forming storage roots were analyzed. Expression was determined by qRT-PCR analyses, using the 2^–ΔCt^ method and phospholipase D1a as reference gene. qRT-PCR data are mean (±SE) of three independent biological replicates (each representing roots pooled from six independent plants). Significance analysis was performed by using Student’s *t*-test (*P* ≤ 0.05), where unlike letters represent significant differences between the different root parts within a group.

Transcript levels of all tested positive regulators (*IbNAC075*, *IbVND7*, and *IbSND2*) were significantly higher in the P compared to the D part of the root, as evident in ARs sampled at 2W after planting (Figure 6A). These differences between the two root parts were found to increase in ARs sampled at 5W after planting (being 60-, 579-, and 3-fold for *IbNAC075*, *IbVND7*, and *IbSND2*, respectively, when the P root part was compared to SRs formed at the D part; Figure 6A). The expression level of the negative regulator *IbXND1* was, however, 2.5-fold lower in the P root part compared to the D part when sampled at 5W (Figure 6B). Expression of *IbVNI2-like* was found to be the highest in 5W SRs formed at the D part (Figure 6B).

**FIGURE 7:**
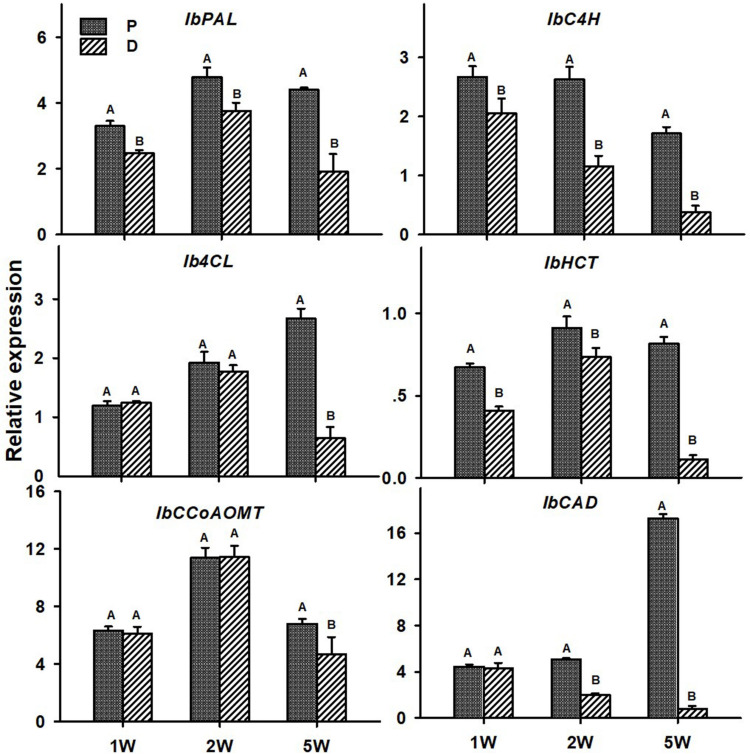
Expression profiles of Sweetpotato “*Georgia Jet*” orthologs of lignin-biosynthesis genes in proximal (P) and distal (D) parts of the adventitious root during development. Transcript levels were measured in roots collected at 1, 2, and 5 weeks after planting (1W, 2W, and 5W, respectively). At 5W, roots forming storage roots at D part were analyzed. Expression was determined by qRT-PCR analyses, using the 2^–ΔCt^ method and phospholipase D1a as a reference gene. qRT-PCR data are mean (±SE) of three independent biological replicates (each representing roots pooled from six independent plants). Significance analysis was performed by using Student’s *t*-test (*P* ≤ 0.05), where unlike letters represent significant differences between the different root parts within a group.

As for lignin-biosynthesis genes, transcript levels of the following genes were tested in the P and D parts of the AR, at 1W, 2W, and 5W after planting: *IbPAL*, *IbC4H*, *Ib4CL*, *IbHCT*, *IbCCoAOMT*, and *IbCAD*. These genes/orthologs were chosen from a database of lignin-biosynthesis genes that were previously identified by us in the sweetpotato root transcriptome (comparing between initiating SRs and lignified roots of the same age), pointing to the presence of multigene families (see Table 5 in Firon et al., 2013). The choice of genes for the current study was based on a previous study where expression of these genes correlated nicely with lignin accumulation (Singh et al., 2019). All tested sweetpotato lignin-biosynthesis genes exhibited significantly higher expression (more than 2-fold; except for *IbCCoAOMT* that exhibited 1.4-fold higher expression) in the P part of the AR compared to the D part, when sampled at 5W after planting (Figure 7). The following genes exhibited higher expression in the P part, as compared to the D part, as early as 1W after planting: *IbPAL*, *IbC4H*, and *IbHCT*. Expression of *IbPAL, IbC4H, IbHCT*, and *IbCAD* was found to be significantly higher in the P part (1. 3-, 2. 3-, 1. 2-, and 2.5-fold higher than in the D part, respectively) as early as 2W after planting (Figure 7).

**FIGURE 8:**
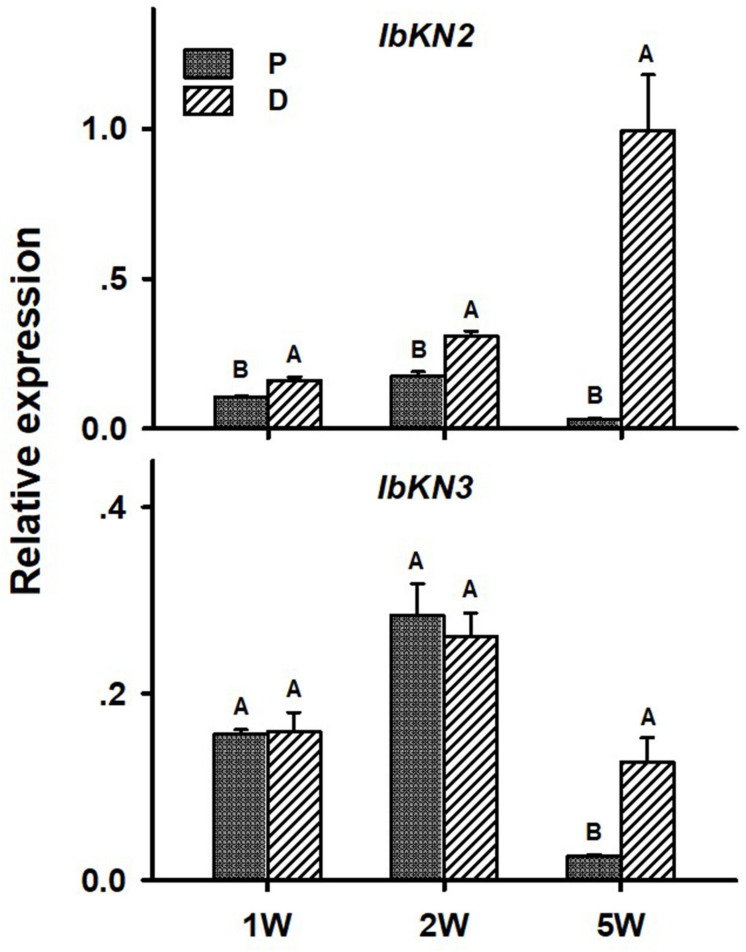
Expression profiles of Sweetpotato “*Georgia Jet*” orthologs of *class I knotted 1-like* (*IbKN2*, *3*) genes in proximal (P) and distal (D) parts of the adventitious root during development. Transcript levels were measured in roots collected at 1, 2, and 5 weeks after planting (1W, 2W, and 5W, respectively). At 5W, roots forming storage roots at the D part were analyzed. Expression was determined by qRT-PCR analyses, using the 2^–ΔCt^ method and phospholipase D1a as reference gene. qRT-PCR data are mean (±SE) of three independent biological replicates (each representing roots pooled from six independent plants). Significance analysis was performed by using Student’s *t*-test (*P* ≤ 0.05), where unlike letters represent significant differences between the different root parts within a group.

In the context of lignification regulation, it was interesting to test the transcript profiles of two class I knotted 1-like (*KNOX*) transcription factors *IbKN2* and *IbKN3*, which are orthologs of the *Arabidopsis BP* gene. *KNOX* genes were previously found, in other systems, to regulate meristem/cambium activity, on one hand, and cause lignin biosynthesis down-regulation, on the other hand (Mele et al., 2003), thus serving as good candidates for regulating the developmental switch of the AR into a SR. In addition, *BP* orthologs were found to be highly expressed in the sweetpotato initiating SR transcriptome (Firon et al., 2013). Expression of both *IbKN2* and *IbKN3* followed a similar pattern when tested in roots sampled at 5W after planting, exhibiting significantly higher transcript levels in the D part of the root (Figure 8). The expression level of *IbKN2* was higher in the D as compared to the P part of the root, already at 1W and 2W after planting (Figure 8).

**FIGURE 9:**
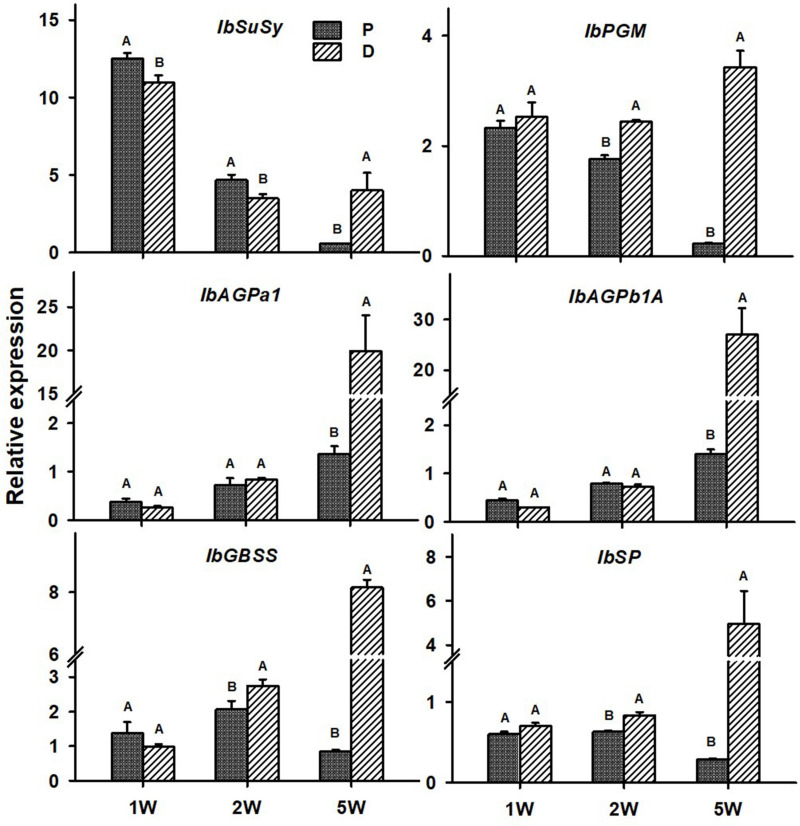
Expression profiles of Sweetpotato “*Georgia Jet*” orthologs of carbohydrate metabolism and starch biosynthesis genes in proximal (P) and distal (D) parts of the adventitious root during development. Transcript levels were measured in roots collected at 1, 2, and 5 weeks after planting (1W, 2W, and 5W, respectively). At 5W, roots forming storage roots at the D part were analyzed. Expression was determined by qRT-PCR analyses, using the 2^–ΔCt^ method and phospholipase D1a as reference gene. qRT-PCR data are mean (±SE) of three independent biological replicates (each representing roots pooled from six independent plants). Significance analysis was performed by using Student’s *t*-test (*P* ≤ 0.05), where unlike letters represent significant differences between the different root parts within a group.

The following six sweetpotato genes, known to be involved in carbohydrate metabolism and starch biosynthesis, were studied for their expression behavior in the P and D parts: *IbSuSy*, *IbPGM*, *ADP-glucose pyrophosphorylase* small and large subunits (*IbAGPa1* and *IbAGPb1A*, respectively), *IbGBSS*, and *IbSP* and results are presented in Figure 9. Expression of all tested genes was elevated in the D part of the root (more than sevenfold), as compared to the P part, when tested at 5W after planting (Figure 9). At this stage of development, the D part of the root showed obvious signs of SR formation (bulking). The *IbPGM*, *IbGBSS*, and *IbSP* genes exhibited higher expression levels in the D compared to the P part already at 2W after planting (Figure 9).

Interestingly, at 5W after planting, there were significant differences in transcript levels of the positive regulators (*IbNAC075* and *IbVND7*), lignin biosynthesis genes (*IbC4H, IbHCT*, and *IbCAD*), regulator of meristem activity (*IbKN2*), and genes of carbohydrate metabolism and starch biosynthesis (*IbAGPa1*, *IbAGPb1A*, *IbGBSS*, and *IbSP*) between the P and D parts of the root, even in those roots that did not develop SRs (Supplementary Figure 4). These differences followed the same trend/profile as the differences in expression detected between the 5W P part of the AR and the D part that developed into a SR (Figures 6–9, respectively), but were significantly smaller.

## Discussion

Root system architecture parameters were previously indicated to be linked to SR initiation and yield (using different sweetpotato varieties and testing the effect of nitrogen fertilization and virus symptom development; Villordon et al., 2012, 2014; Villordon and Clark, 2014). Such a correlation was further indicated by our recently published results demonstrating that exogenous application of the plant hormone gibberellin caused reduction in most tested RSA parameters, measured during the first 2W after planting (the SR initiation phase), in parallel to significant reduction in SR formation (Singh et al., 2019). In the present study, all tested RSA parameters, including LR number, length, and density, were found to be significantly increased/higher in the D part of the sweetpotato AR as compared to the P part, correlating with the formation of a SR. Interestingly, such an association was already evident at an early stage of root development, 2W after planting, when no visible signs of SR formation were observed (like bulking and change in AR color). Root system architecture differences between the P and D parts of the root increased with development and were high at 5W after planting, when SR formation was obvious. Our results thus support the suggestion of Villordon and Clark (2014) that root architecture is important for root (sweetpotato and cassava) and tuber (potato) crop productivity.

Cassava root system consists of two main types of roots, lignified non-SRs that produce a high number of xylem vessels and fibers and SRs that produce starch-accumulating parenchyma cells (Siebers et al., 2017). In sweetpotato, similar to cassava, we have previously demonstrated that roots that do not develop into SRs exhibit massive formation of xylem vessels and fibers and intensive stele lignification, while the developmental transition into a SR involves the formation of primary and secondary cambial cells that will form starch-accumulating parenchyma cells in the root vascular cylinder (Villordon et al., 2009). Furthermore, external factors like stress conditions or application of gibberellin, which cause enhanced xylem formation and lignification, were shown to affect development of SRs and reduce yield, suggesting that xylem formation and lignification inhibit SR development (Siebers et al., 2017; Singh et al., 2019). It was thus interesting to compare the anatomy of the P and D parts of the sweetpotato root during development, in order to find out whether there are differences in xylem vessels and fiber formation, as well as lignification, between these two parts.

In the current study, we show that, as early as 1W and 2W after planting, there are significant differences in the number of xylem vessels and the area occupied by xylem fibers, between the two root parts, with the P part exhibiting enhanced xylem development together with increased/massive lignin deposition. At the same time, the D part exhibited significantly increased/elevated starch accumulation as compared to the P part. Togari (1950) suggested that lignification inhibits SR formation. Additional work is needed, however, in order to conclude whether the high degree of lignification detected by us at the P part of the root, evident early during development, inhibits its capacity for SR formation, or a yet unidentified upstream signal is involved in the regulation of the developmental fate of the two root parts.

The big question is, what are the mechanisms that enable the developmental change of the D part of the root into a storage organ, and how such mechanisms are regulated at the molecular level. Transcription profiling in initiating SRs, compared to lignified roots of the same age, shows down-regulation of lignin biosynthetic genes, while carbohydrate metabolism and starch-biosynthesis genes were up-regulated (Firon et al., 2013), suggesting the importance of upstream regulatory factors that may control such a balance. In the current study, in accordance with increased xylem development and lignification levels in the P part of the root (as compared to the D part), significantly higher transcript levels of the sweetpotato orthologs of *Arabidopsis NAC075* and *VND7* (*IbNAC075* and *IbVND7*) regulatory genes was demonstrated as early as 2W after planting. In addition, a decrease in expression levels of the potential negative regulators, *IbXND1* and *IbVNI2-like*, was observed in the P part at 5W after planting. These results, together with additional recently published results (Singh et al., 2019), point to *IbNAC075* and *IbVND7* as potential master switches of root xylem proliferation and lignin biosynthesis, suggesting that their down-regulation at the D part of the root causes down-regulation of the lignin-biosynthesis pathway.

Lignin accumulation was demonstrated in numerous studies and systems (carrot, cassava, and sweetpotato root development) and correlated with changes in transcript levels of lignin biosynthesis genes (Zhong and Ye, 2009; Sojikul et al., 2015; Wang et al., 2017; Singh et al., 2019). In the current study, all tested sweetpotato lignin biosynthesis genes exhibited highly increased levels in the P part of the root as compared to the D part, with relative transcript levels of *IbPAL*, *IbC4H*, and *IbHCT* exhibiting an increase as early as 1W after planting. These results correlate with the higher lignin accumulation detected in the P compared to the D root part, during development. Such a correlation between lignin levels and gene expression of lignin-biosynthesis genes was shown by us previously in sweetpotato roots (Firon et al., 2013; Singh et al., 2019). It should be mentioned that at 1W after planting, lignin deposition was still limited, suggesting that the higher expression of *IbPAL*, *IbC4H*, and *IbHCT* (upstream genes in the pathway) might be related to the biosynthesis of phenolic compounds other than monolignols. SRs may contain different phenolics, depending on the variety (Sun et al., 2019).

In parallel to down-regulation of the lignin-biosynthesis pathway, the D part of the root exhibited up-regulation of starch biosynthesis. The *IbKN2* transcription factor (ortholog of the *Arabidopsis KNOX1 BP* transcription factor, regulator of meristem maintenance; Mele et al., 2003) was suggested by us recently as a good candidate for regulating SR development, by operating at an intersection between down-regulation of lignification and enabling up-regulation of root cambium development and starch accumulation (Singh et al., 2019). In accordance with this hypothesis, up-regulated transcript levels of *IbKN2* were observed in the D as compared to the P part, as early as 1W after planting. The increased transcript levels of *IbKN2* in the D part lasted during all tested stages of root development (2W and 5W after planting).

It was interesting to note that, as early as 1W after planting, the D part of the root exhibited significantly higher levels of starch compared to the P part, and that these differences lasted and further increased during 2W and 5W after planting. In accordance with the high levels of starch accumulation observed in the D part of the roots at 5W after planting (in SRs), expression/transcript levels of all tested carbohydrate metabolism genes were elevated in the D part as compared to the P part. The expression of key starch-biosynthesis genes, *IbPGM IbGBSS*, and *IbSP* (Geigenberger, 2011), was already up-regulated in the D part of the root at 2W after planting, pointing to their involvement in starch accumulation in the developing sweetpotato SR.

The results, showing differences in xylem development and fiber formation as well as gene expression of vascular regulators and lignin biosynthesis genes, at 5W after planting, between the P and D parts of the root, even in those roots that did not develop SRs, further indicate that the P and D parts of the AR exhibit a different developmental program. These results (together with gene expression results of *IbKN2* and starch biosynthesis genes) raise the possibility that SR initiation is at least a three-stage developmental program exhibiting, at the first stage, down-regulation of secondary growth and lignin biosynthesis, dictated by down-regulation of vascular regulators and lignin biosynthesis genes. This is followed by stage 2, characterized by cambium cell proliferation being regulated by another gene or a set of genes. The following stage, stage 3, involves the formation of a large number of starch-accumulating parenchyma cells. Furthermore, these milder/smaller differences between P and D parts, in non-SRs as compared to SRs, suggest the importance of the level of expression that needs to cross a specific threshold in order to initiate the storage-organ developmental program.

**FIGURE 10:**
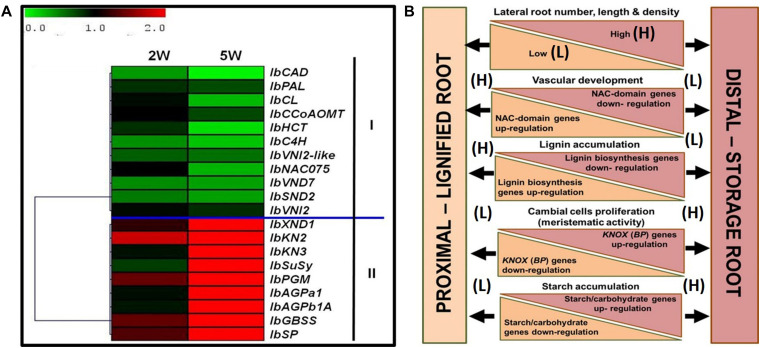
Summary of the main differences between the proximal (P) and distal (D) parts of the sweetpotato adventitious root that mark the developmental change of the D part into a storage root. **(A)** Heat map and hierarchical clustering representing the relative expression profiles of genes involved in regulation of vascular development, lignin biosynthesis, meristematic activity, and carbohydrate metabolism. The hierarchical clustering is based on Pearson’s correlation, which allows gene clustering according to their expression pattern and level. Relative expression of genes in D part as compared to P part was determined at 2W and 5W after planting by the 2^–ΔΔCt^ method, using phospholipase D1a as a reference gene. The green and red color corresponds to low and high relative expression, respectively. Genes were clustered into two groups (I and II), containing down-regulated and up-regulating genes, respectively. **(B)** Schematic presentation of the main differences between P and D parts of the adventitious root during development, pointing to higher lateral root number, length, and density in the D part as compared to the P part of the root, together with a decrease in xylem fiber formation and increased meristematic activity/cambial cell formation. These differences come together with down-regulation of genes related to lignin biosynthesis (*IbPAL, IbC4H, Ib4CL, IbCCoAOM*, and *IbCAD*) and NAC domain (*IbNAC075, IbVND7*, and *IbSND2*), and up-regulation of *KNOX* (*IbKN2* and *IbKN3*) and carbohydrate metabolism/starch biosynthesis (*IbSuSy, IbPGM, IbAGPa1, IbAGPb1A, IbSP*, and *IbGBSS*) genes.

Figure 10 summarizes the main differences between the P and D parts of the sweetpotato AR, marking the developmental change of the D part into a SR. The findings highlight specific markers as well as potential regulators of SR formation in sweetpotato. A heat map and hierarchical clustering, relating to the relative expression profiles of genes involved in regulation of vascular development, lignin biosynthesis, meristematic activity, and carbohydrate metabolism, are given, emphasizing the major processes taking place in the D part of the root at an early phase of root development. The resulting picture highlights down-regulation of lignin-biosynthesis genes and their potential upstream regulators, on one hand (group I), and up-regulation of starch-related genes and the *KNOX1* transcription factor, on the other hand (group II), as the main events underlying the development of the D part into a storage organ (Figure 10A). A model is suggested (Figure 10B), pointing to the main factors that play a role in SR formation, serving as a basis for further studies. Comprehensive transcriptome and metabolome studies, including plant hormone analyses, comparing between the P and D root parts during development, are suggested for shedding light on the regulation of this important process of SR formation. Gibberellin may be a good candidate for regulating root lignification levels (Singh et al., 2019), while both cytokinins and auxin may be involved in promoting cambial activity and cell division (Ravi et al., 2014) needed for SR development.

## Data Availability Statement

All data generated or analyzed for this study are included in the published article and in the Supplementary Material (Supplementary Figures 1–4 and Supplementary Table 1).

## Author Contributions

VS and NF conceived and designed the experiments, analyzed the data, and wrote the manuscript. VS performed all the experiments. HZ helped in anatomical analyses. SS provided technical assistance in starch analyses. RA helped in interpretation of the anatomical data, vessel formation, and lignification. JY and PZ critically discussed the experiments and the results, and reviewed the manuscript. LS and WL participated in evaluating the experimental design and results. NF critically reviewed the manuscript. All authors discussed the results and approved the manuscript.

## Conflict of Interest

The authors declare that the research was conducted in the absence of any commercial or financial relationships that could be construed as a potential conflict of interest.

## Footnotes

1 http://www.bioinformatics.nl/cgi-bin/primer3plus/primer3plus.cgi

2 http://mev.tm4.org

3 http://planttfdb.cbi.pku.edu.cn/

## Abbreviations

AGPase: ADP glucose pyrophosphorylase
AR: adventitious root
*BP*: *BREVIPEDICELLUS*
CAD: cinnamyl alcohol dehydrogenase
CCoAOMT: caffeoyl-CoA O-methyltransferase
CCR: cinnamoyl-CoA reductase
C3’H: p-coumaroyl shikimate 3’-hydroxylase
C4H: cinnamic acid 4-hydroxylase
4CL: 4-coumarate:CoA ligase
COMT: caffeic acid/5-hydroxyferulic acid O-methyltransferase
D: distal
F5H: ferulate 5-hydroxylase
GBSS: granule-bound starch synthase
HCALDH: hydroxycinnamaldehyde dehydrogenase
HCT: hydroxycinnamoyl-coenzyme A shikimate:quinate hydroxycinnamoyl-transferase
KNOX: Class I knotted 1-like
LR: lateral root
NST/SND: NAC secondary-wall thickening promoting factor/secondary-wall associated NAC domain protein
P: proximal
PAL: phenylalanine ammonia-lyase
PGM: phosphoglucomutase
Ph-HCl: phloroglucinol-HCl
RSA: root system architecture
SP: starch phosphorylase
SR: storage root
SuSy: sucrose synthase
VND: vascular-related NAC domain
VNI2: VND-INTERACTING2
XND1: XYLEM NAC DOMAIN 1.
