# Supplementary material for: Proximal and Distal Parts of Sweetpotato Adventitious Roots Display Differences in Root Architecture, Lignin, and Starch Metabolism and Their Developmental Fates
Source: Front Plant Sci. 2021 Jan 21;11:609923. doi: 10.3389/fpls.2020.609923 (PMC7855870; doi:10.3389/fpls.2020.609923)
Supplement: Supplementary file 1 [file Data_Sheet_1.PDF]

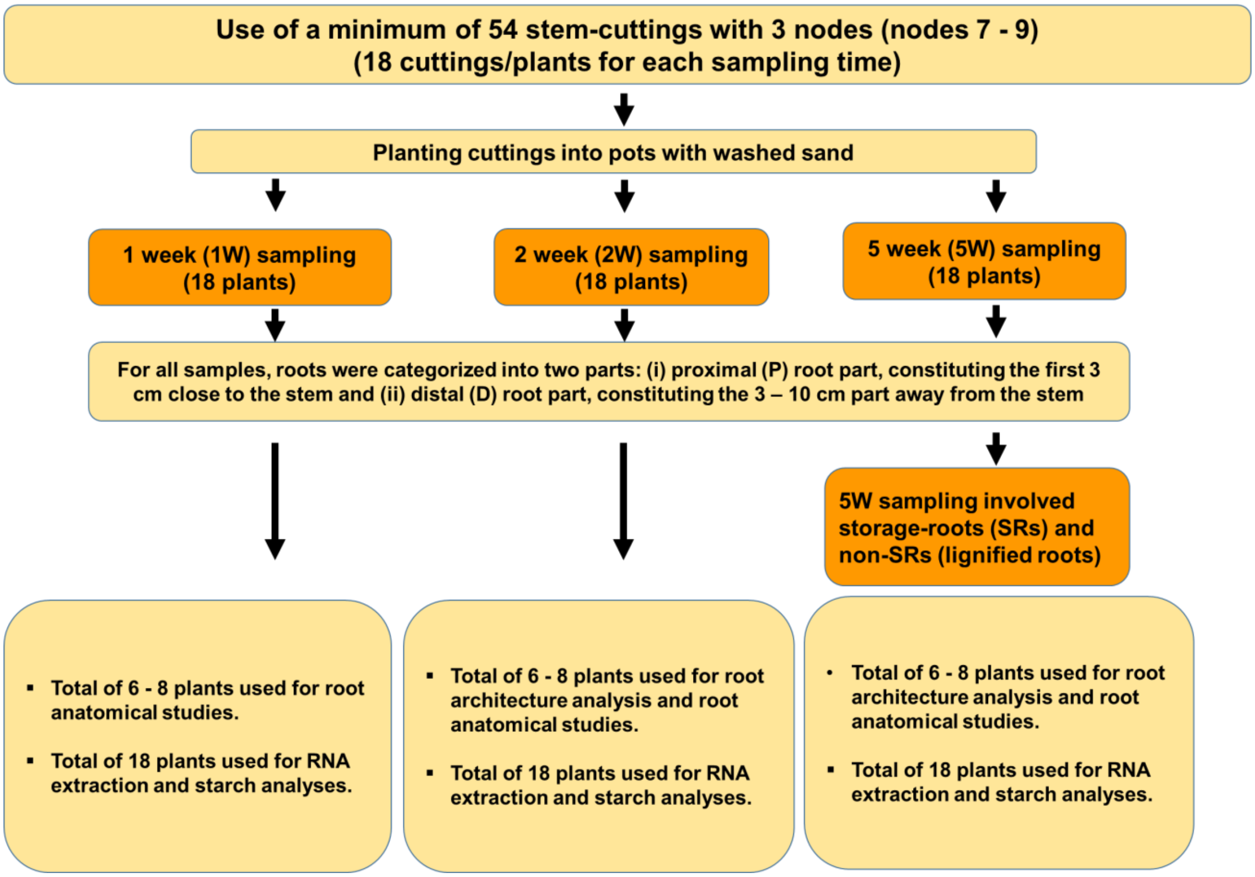

Supplementary Fig. S1. Schematic representation of the experimental setup. Details are given in 'Materials and methods' section. For anatomy, roots were sampled in FAA. For RNA extraction and starch analyses, roots were immediately frozen at - 80 ^{o}C. Root system architecture (RSA) parameters included lateral root (LR) number per plant, LR cumulative length per plant, and LR density per adventitious root. For RSA and root anatomy, 6 – 8 plants were sampled (using one root per plant). The 5 week sampling, included both, storage-roots and non-storage roots. For RNA extraction and starch analyses, 3 independent biological replicates (each representing roots pooled from 6 independent plants) were used. A total of 18 plants were used per each sampling time for all analyses (RSA, anatomy, starch, gene expression) as indicated above.

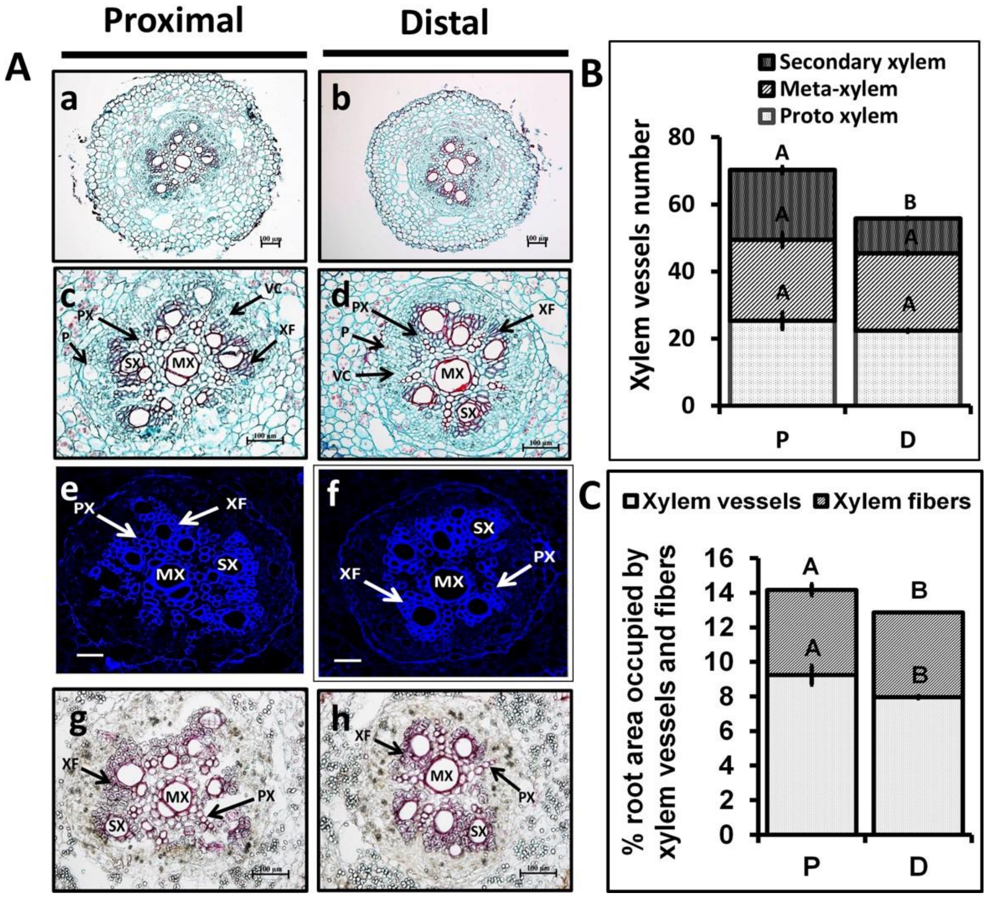

Supplementary Fig. S2. Differences between proximal and distal parts of Sweetpotato "*Georgia Jet*" adventitious roots, sampled at five weeks after planting, in those cases where the distal part did not develop into a storage-root. Adventitious roots were sampled at 5 weeks after planting. Proximal (P) and distal (D) parts of the root were sampled at 0 – 3 and 3 – 10 cm from the stem, respectively. Anatomy of the root is presented in (**A**). Sections were stained with safranin and fast green (a, b, c, d), analysed by auto-fluorescence imaging (e, f) or stained with phloroglucinol-HCl (g, h). Sections represent 6 - 8 roots sampled from individual plants. PX, protoxylem; MX, metaxylem; SX, secondary xylem; XF, xylem fibers; P, phloem; VC, vascular cambium. Scale bar = 100 µm (a, b, c, d, g, h), and 50 µm (e, f). Xylem vessel number and percent (%) root area occupied by xylem vessels and fibers in P and D parts of the adventitious roots are presented in (**B**) and (**C**), respectively. Recorded xylem vessels included protoxylem, metaxylem and secondary xylem. Bars represent mean of 6 - 8 roots sampled from individual plants ± SE. Significance analysis was performed by using student's t-test (*$P \leq 0.05$)*, where unlike letters represent significant differences between the two root parts within a sampling group.

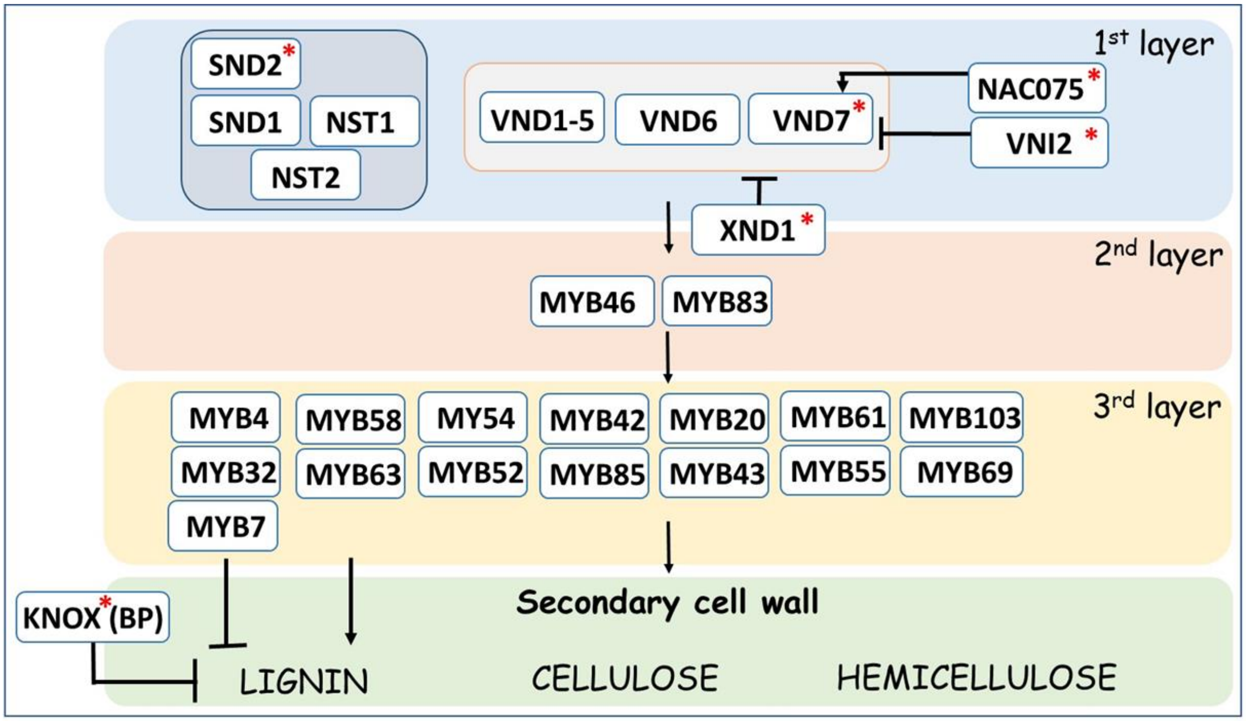

Supplementary Fig. S3. Schematic representation of the molecular regulation of secondary vascular development and secondary wall formation marking the genes used in the current study. The presented scheme is a modification of the scheme presented by Zhang et al. (2018). For identifying the respective sweetpotato genes/orthologues, we used the *Georgia Jet* root transcriptome database (Firon et al., 2013) and homology searches that were done using Blast2GO, non-redundant NR and the plant TFDB databases (http://planttfdb.cbi.pku.edu.cn/). None of the second level orthologues (MYB46/83) or downstream transcription factors (such as MYB58/63, for example) were detected, except five MYB4-like orthologues that did not give reproducible results. An orthologue of the class 1 knotted-like homeobox (KNOX1) gene is included in the scheme, homologous to the BP gene shown to down-regulate lignin biosynthesis in *Arabidopsis* (Mele et al., 2003), used as a marker gene for cambium cells proliferation during sweetpotato storage-root initiation (Firon et al., 2013). * indicates the genes investigated in present study.

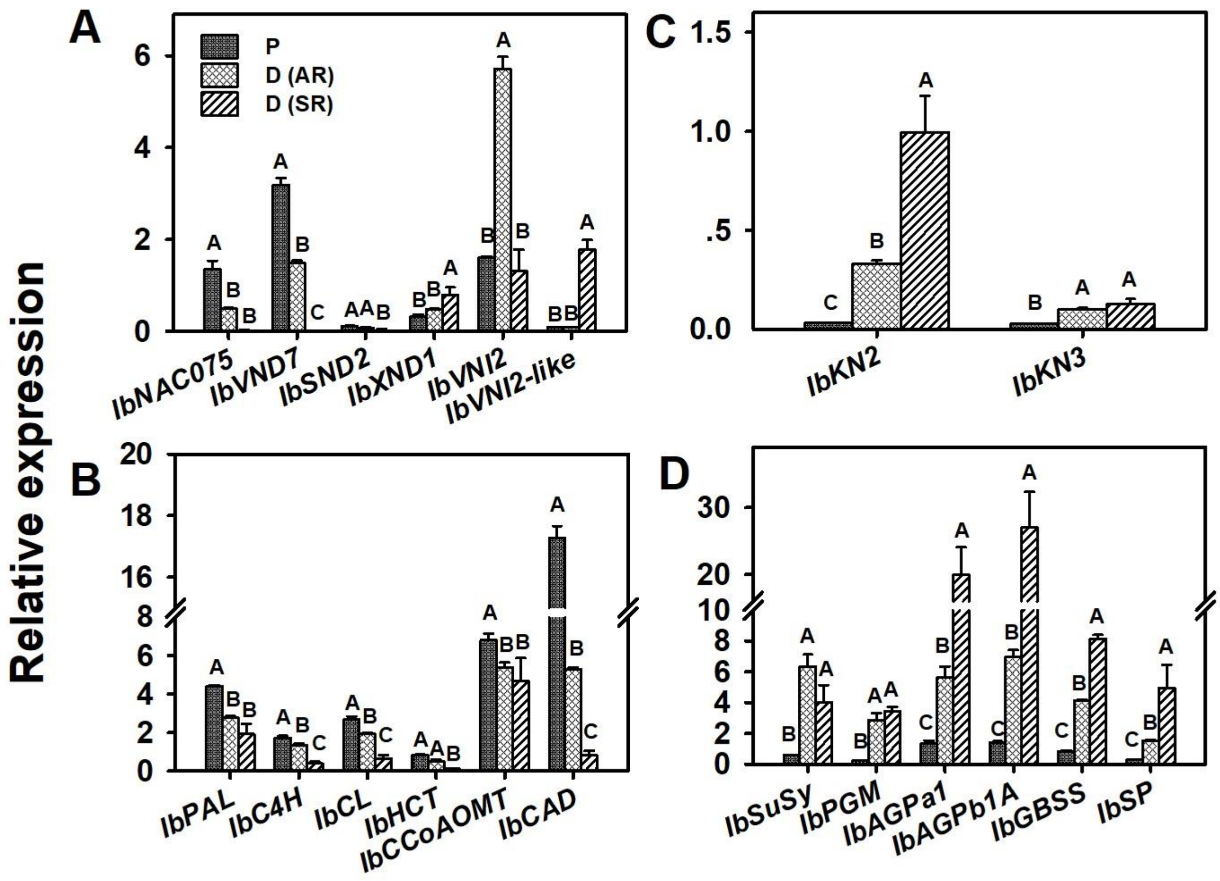

Supplementary Fig. S4. Expression profiles of Sweetpotato "*Georgia Jet*" orthologues of potential positive and negative regulators of vascular development (A), lignin-biosynthesis genes (B), *class I knotted 1-like* (*IbKN2*, *3*) genes (C) and carbohydrate metabolism and starch biosynthesis genes (D), in proximal (P**) and distal parts (**D**) of the adventitious root at five weeks after planting**. The distal part analyzed was either a storage root (SR) or a non-storage-root (AR). Expression was determined by qRT-PCR analyses, using the $2^{-\Delta Ct}$ method and phospholipase D1a as reference gene. qRT-PCR data are mean (± SE) of three independent biological replicates (each representing roots pooled from six independent plants). Significance analysis was performed by using student's t-test ($P \leq 0.05$), where unlike letters represent significant differences between the different root parts within a group.
