# Supplementary material for: Proximal and Distal Parts of Sweetpotato Adventitious Roots Display Differences in Root Architecture, Lignin, and Starch Metabolism and Their Developmental Fates
Source: Front Plant Sci. 2021 Jan 21;11:609923. doi: 10.3389/fpls.2020.609923 (PMC7855870; doi:10.3389/fpls.2020.609923)
Supplement: Supplementary file 2 [file Table_1.DOCX]

**Supplementary Table S1.** Primers used for qRT-PCR analysis.

| ***Ipomoea batata* contigs** | ***Ipomoea batata* gene name** | **Forward primer** | **Reverse Primer** |
| --- | --- | --- | --- |
| S_PBL_c36855 | VASCULAR RELATED NAC-DOMAIN PROTEIN 075 (*IbNAC075*) | AAGAGCATCAGCTGTGTGGA | CAATTCTTGATCTGTTGGGTCA |
| S_PBL_c32341 | VASCULAR RELATED NAC-DOMAIN PROTEIN 7 (*IbVND7*) | TAGAATGGAGCCATGGGACA | AGTTCCCGTCGGATACTTCC |
| S_PBL_c24252 | SECONDARY WALL-ASSOCIATED NAC DOMAIN 2 (*IbSND2*) | GCCTGGTCTACCCGCCG | AGGATGAAGTTTCCGGCTGT |
| S_PBL_c504 | XYLEM NAC DOMAIN1 (*IbXND1*) | ACGCTTTTTACGTCGGAGAG | TGGAAGCGGAATAATCGGAGAG |
| S_PBL_c4628 | VND-INTERACTING2 (*IbVNI2*) | ACCCATACCCATACCCATCTTC | AACTCAACTTCTCCATAACCACC |
| S_PBL_c17476 | VND-INTERACTING2-like (*IbVNI2-like*) | ACCCTAGCTCAGGCTCAGAC | CCCTCGCCAAGAAGTCGTAG |
| S_PBL_c2312 | Phenylalanine ammonia lyase (*IbPAL*) | GGATCCAAGAGTGCAGGTCC | CCTTGTCACATTCCTCCCCG |
| S_PBL_c7605 | Cinnamate 4-hydroxylase (*IbC4H*) | GCGGCAAGAAGTACAAGCTC | CTTGGCGTAATCGGTGAGAT |
| S_PBL_c18044 | 4-Coumarate-CoA ligase (*Ib4CL*) | CTGAGGATGAAGTTAAAGAGTTTGTG | GCCTGAGGGAGACTTTGGA |
| S_PBL_c17752 | Hydroxycinnamoyl transferase (*IbHCT*) | CCGTCGCTTACAGCTCCTAC | CGGTGGCTATGTACAGCTTG |
| S_PBL_c2944 | Caffeoyl-CoA-O-methyltransferase (*IbCCoAOMT*) | GAGGCACCCACAAGACTACG | TGGTTGTCTGATTCTCCGCC |
| S_PBL_lrc53688 | Cinnamyl alcohol dehydrogenase (*IbCAD*) | GTCTTGGCGCAGACTCTTTC | TAATGGCACAACAGCGTGAT |
| S_PBL_c8137 | Class I knotted 1-like homeobox (KNOX1) (*IbKN2*) | GCCAGGCAGAAGTTGCTTAG | CAGTGCCGTTTTCTTTGGTT |
| S_PBL_c31412 | Class I knotted 1-like homeobox (KNOX1) (*IbKN3*) | CGCCTAGGTCCATAATCC | TATTTCAAGGCGGTCTCA |
| S_PBL_c543 | Sucrose synthase (*IbSuSy*) | TACTGCATCCTTTCCCAAGC | AGCCATGCTCTCCTTGTCAT |
| S_PBL_c20112 | Phosphoglucomutase (*IbPGM*) | TGAGAAGGATGCTTCCAAGA | GCCAGTGAATTCCTGCATCT |
| S_PBL_c18129 | ADP-glucose pyrophosphorylase alpha subunit (*IbAGPa1*) | CAATTGGAATTGGCAGGAAT | TTTCCCTAGCTGCTTCTTGAAC |
| S_PBL_c54187 | ADP-glucose pyrophosphorylase beta subunit (*IbAGPb1A*) | GATTGGGTTAAAGACGTTGTCATC | CACGAATGGTTGCTTTCTCC |
| S_PBL_c3042 | Granule-bound starch synthase (*IbGBSS*) | GGACGTGCTGAAGGTGATAA | CGAGAGCTCTTGTGACATGC |
| S_PBL_c1370 | Starch phosphorylase (*IbSP*) | GTGTGACACCAAGAAGATGGA | CGCAGTTCTGCCAACTTTTC |
| JX177360.1 | Phospholipase D1a (*IbPLD*) | ATCGGAATCAGCAGTGATGG | ATGATGAGGCAAGCAGTGTG |
